# An Overview of the Components of AW-IPM Campaigns against the New World Screwworm

**DOI:** 10.3390/insects3040930

**Published:** 2012-10-12

**Authors:** Thiago Mastrangelo, John B. Welch

**Affiliations:** 1 Universidade de São Paulo / Avenida Centenário 303, 13400-970, Piracicaba, SP, Brazil; 2 USDA-APHIS Screwworm Eradication Program / 9100 Panama Place, Washington, D.C. 20521, USA; E-Mail: John.B.Welch@aphis.usda.gov

**Keywords:** livestock pests, myiasis, *Cochliomyia*, pest management

## Abstract

The New World Screwworm, *Cochliomyia hominivorax* (Coquerel), is one of the most damaging parasites of livestock, causing millions of dollars in annual losses to producers. The fly is an obligate parasite of warm-blooded animals, including humans. After a successful 50-year eradication campaign, *C. hominivorax* has been eradicated from the USA, Mexico and Central America by an area-wide integrated pest management approach. Recently, Caribbean and South American countries have expressed an interest in this approach. Aiming to support forthcoming projects in these countries, this review describes the main technical components of past and ongoing AW-IPM campaigns against *C. hominivorax*.

## 1. Introduction

The New World Screwworm (NWS), *Cochliomyia hominivorax* (Coquerel, 1858) (Diptera: Calliphoridae), is an obligate parasite of warm-blooded animals, causing primary myiasis in pre-existing wounds. The fly has a very high reproductive rate and females can oviposit up to 450 eggs at 3–4 days intervals on the dry edge of wounds or body orifices. The eggs hatch after 12–20 hours, the larvae migrate immediately to the wound and start feeding on the wound fluids. The second and third instar larvae burrow deeply into the host tissue for feeding. Mature larvae leave the animal and pupate in the soil and the entire life cycle can be a short as 20 days under optimal environmental conditions [1,2,3].

The larvae from a single oviposition can kill smaller animals and multiple infections can kill mature cattle [4,5,6]. Other consequences of screwworm infestations are increased costs to the producer with veterinary services, labor with surveillance and animal management. Additional losses include decrease in animal weight, wool, mohair, and milk production. According to Baumhover *et al.* [4], the estimated losses in southeastern and southwestern USA were *ca.* US$ 20 million/year and US$ 50–100 million/year, respectively. In 1935, an infestation with 230,000 myiasis cases was reported in USA [7] and Parish [8] reported 6,148 cases during the screwworm seasons from 1936 to 1940 in Texas. Vargas-Teran *et al.* [9] estimated the losses in US$ 1.7 billion/year in Brazil alone.

Humans are also hosts and the literature contains numerous reports of human infestations in Colombia, Suriname, Guyana, Ecuador, Paraguay, Peru, Bolivia and Uruguay. Leão [10] described 51 traumatic myiasis cases in the Amazon basin, and a 59 year old woman was found being eaten alive by screwworms in the rural zone of Belém, Brazil. Coronado & Kowalski [11] reported 241 cases of infants and elderly people in Venezuela, with two deaths as a consequence of massive infestation.

The NWS was originally distributed throughout South and Central America, the southwestern States of USA and across the tropical belt to Florida [6,12,13]. However, after a successful 50-year eradication campaign, the NWS had been eradicated from the USA and Central America by an area-wide integrated pest management (AW-IPM) approach and this pest is currently present only in parts of the Caribbean (Cuba, Dominican Republic, Haiti, Jamaica, Trinidad and Tobago) and South America (except Chile) [14]. 

An IPM approach can be defined as “a decision support system for the selection and use of pest control tactics, singly or harmoniously, based on cost/benefit analyses that take into account the interests of and impacts on producers, society, and the environment” [15]. The AW-IPM concept is used when the IPM approach is applied against an entire pest population within a large predefined geographic area with the objective of eradicating or to maintain pest population levels below an economic threshold [16]. Trapping or monitoring procedures, correct taxonomic identification of pests, and selection and use of control methods are the tactical components of any AW-IPM campaign. Aiming to support forthcoming projects in Caribbean and South America, this review describes the main technical components (e.g., traps, attractants, animal management, pesticides, and sterile insects, among others) of AW-IPM campaigns against *C. hominivorax*. 

## 2. Morphological Identification of Primary and Secondary NWS Fly

The genus *Cochliomyia *Towsend is comprised of four Calliphoridae species in the Neotropical region, *Cochliomyia aldrichi *(Del Ponte, 1938), *C. minima* (Shannon, 1926), *C. hominivorax *(Coquerel, 1858) and *C. macellaria *(Fabricius, 1775) [17]. *C. aldrichi *and *C. minima* are restricted geographically to some Caribbean islands and Florida [18]. Their immature stage is still undescribed, and a single case of *C. minima* myiasis has been reported in Puerto Rico [19].

*C. macellaria*, the secondary screwworm, is the species most commonly confused with *C.hominivorax* in myiasis. This fly is a sarcosaprophagous species, but can be found as a secondary invader in wounds. It is distributed from southern Canada to Argentina [18]. The geographical overlap and the morphological similarity between *C. macellaria *and *C. hominivorax *can lead to misidentification by non-specialists, but some larvae and adult morphological characters are reliable to distinguish the two species [20]. 

*C. hominivorax *third-instar larvae can be easily distinguished due to the presence of heavily pigmented tracheal trunks from posterior spiracles (up to segment 9 or 10) and 7–12 papillae in the anterior spiracle. In *C. macellaria*, the tracheal trunks are not heavily pigmented, and dorsal spines are not found in the posterior margin of segment eleven [20]. 

In *C. hominivorax* adults, black setulae can be found in the parafacialia of the head. The central stripe on thorax extends just forward of mesonotal suture. The fifth segment of abdominal tergite is usually very slight dusting laterally. In females, the basicostal scale is dark brown to almost black [20].

In the fronto-orbital plates of *C. macellaria *adults, only yellow hairs can be found (but their insertions appear as black dots). The central stripe on thorax extends well forward of mesonotal suture. The fifth abdominal tergite presents dense white spots laterally. In females, the basicostal scale is yellow [20]. 

Sex identification of adults can easily be carried out for both species as males are holoptic or subholoptic and females are dichoptic [18]. The sexual dimorphism is usually magnified in *C. hominivorax *[21]. 

Wing morphometry can be another tool for identification. Lyra *et al.* [21] described a simple method to identify *C. hominivorax* and *C. macellaria* through wing morphometrics, suggesting that the partial warp scores could be used as basis of a test to distinguish both species. The major disadvantage of a wing shape test is that only non-damaged wings can be used. 

Different methods based on molecular markers for the identification of *C. hominivorax* and *C.macellaria* have also been investigated with success [21,22,23]. 

## 3. Survey Techniques

Egg masses collected from sentinel animals, submission of larval samples from wounds by the public and animal health authorities, and collection of adults using trapping systems are essential in surveillance for NWS populations and for monitoring the progress of any AW-IPM campaign against *C. hominivorax*.

Larvae can be obtained through regular inspection of livestock and wild animals. After being pulled from the wound with forceps, larvae can be dropped directly into 100% ethanol (for molecular or genetic studies) or put in a sealed container for later processing. To extend and to prevent them from turning black, larvae can be killed by immersion in hot water (> 80 °C) for 15–30 s and then stored in formalin or 80%–100% ethanol at ambient or lower temperatures. Alternatively, live larvae can be fixed in Kahle’s fixative for up to 24 h, rinsing in water and stored in 80%–100% ethanol [20].

Wounded sentinel animals, especially sheep or goats, have been used in research and eradication campaigns to obtain egg masses. Female flies are attracted to infested wounds’ odors resultant from destruction and necrosis of tissues by larvae and bacteria [2,24,25]. If not enough “naturally” wounded animals are available (*e.g*., animals exposed to ticks, horn and stable flies, animals undergoing husbandry procedures, neonatal calves and lambs), consideration should be given to deliberate wounding of sentinels. The cost of sentinel animals, their intensive care, difficulty to get approval from animal welfare authorities, problems in transporting and keeping them in diverse habitats are factors that favor alternate methods of attracting adults. Parker & Welch [26] suggested another method to obtain egg masses, which consisted in collecting females from rotted liver, holding them for three days and then providing stimuli to oviposit. This method is easier, cheaper, takes less time, and feasible in developing countries and even under primitive working conditions. 

A few trapping systems are available for collecting adults. Bishopp [27] was the first to recommend the use of traps to survey blowflies. Subsequently, other researchers started using decomposing meat or meat by-products in water as baits. In the 1930s, Laake & Cushing [28] used one trap baited with decomposing liver per km^2^ in central Texas [29] to capture screwworm flies. During the next 40 years, the Bishopp trap (with only slight modifications) baited with moistened beef liver was used as standard trapping system for the NWS. In the 1970s, several attempts to find another attractant were made. Grabb & Turner [30] isolated the attractive components derived from incubated blood tested by DeVaney *et al.* [31] and that were similar to decomposing animal wounds.

Under field conditions, Jones *et al.* [32] continued the research and selected 10 of the more attractive compounds (acetic acid, benzoic acid, butyric acid, valeric acid, phenol, *p*-cresol, indole, *iso*-butyl alcohol, *sec*-butyl alcohol, and acetone). The new chemical bait (the first “*Swormlure*”) was as attractive as liver to NWS adults and much less attractive to other blowfly species, reducing significantly the time required to identify trap catches [32]. Other advantages over liver were that the new mixture was cleaner and easier to work with, and it was more uniform in attractiveness because environmental variations that influence the type of flora associated with liver were no longer a factor.

Coppedge *et al.* [33] refined the *Swormlure* by mixing the compounds in different proportions, adding methyl disulfide and eliminating acetone. The new attractant, called *Swormlure*-2, proved to be more attractive to NWS than the original mixture in Bishopp traps and as attractive as decomposing liver. The capture of non-target flies also remained low. *Swormlure*-2 has a strong odor which permeates almost any material (except metal). Trials conducted at the Moore Air Base, Texas, indicated that the instantaneous area of influence of *Swormlure*-2 extended 125–150 m downwind of the attractant source [34].

Once mixed, *Swormlure*-2 has a shelf life of more than three months [35]. Brown & Mackley [36] provided a good understanding of the changes on attractancy and chemical composition under field conditions, allowing the prediction of effective life of *Swormlure*-2 in trapping systems in the field. Dimethyl disulfide, butanol, butyric acid, and acetic acid appeared to evaporate the most rapidly. On the other hand, phenol, valeric acid, *p*-cresol, benzoic acid, and indole exhibited low but constant rates of evaporation. *Swormlure*-2 was more attractive during the first 7 days of exposure. Loss of attractancy appeared to result from changes in relative composition with time of exposure, but sex ratios of fly collections were not affected. Temperature and wind were the main weather-related factors affecting evaporation [36].

Polyester fiber wicks coated with a propylene skin proved to be superior to cotton dental wicks as a slow release mechanism for the *Swormlure*-2 [37]. Their use in reservoir bottles of survey traps reduced the quantity of *Swormlure*-2 released by 10-fold without reducing the number of flies captured.

In the 1980s, as the eradication campaign moved southward from USA into Mexico, the performance of *Swormlure*-2 became increasingly erratic, particularly in humid, tropical areas [38]. The cases of its ineffectiveness demonstrated the need to improve the attractant. Mackley & Brown [38] then evaluated 15 mixtures with the 10 components of the *Swormlure*-2. The most attractive mixture (*Swormlure*-4) contained higher amounts of dimethyl disulfide, butanol, and acetic acid, becoming almost twice as volatile as *Swormlure*-2. Traps baited with the new mixture captured 81% more wild *C. hominivorax* than traps baited with *Swormlure*-2 during 7 days of exposure, but sex ratios for captured flies were similar [38]. 

Since its formulation [33], the *Swormlure*-2 had been used more often in a collapsible trap, which was a modification of the Bishopp blowfly trap [27], by placing about 30 mL of the attractant in two 60 mL vials in the bottom of a large plastic fan that had been used for decomposing liver. A piece of nº 2 cotton dental roll extending 1.3 cm above the mouth of the bottle was provided as evaporation surface. This collapsible trap was basically a cylinder (66 cm high x 41 cm in diameter) of plastic screen with a vertical funnel attached to the bottom rim of the cylinder. Flies attracted to the bait were caught as they moved up inside the funnel, supposedly toward the light. This trap was considered inefficient since numerous flies crawled over the attractant container, but did not move up the funnel [39].

A few months after the development of *Swormlure*-2, Broce *et al.* [40] developed a wind oriented trap (WOT) which swung freely and maintained nearly constant orientation with the attractant upwind by wind vanes (design and assembly were included in their study). *Swormlures* could be deployed in two bottles with dental cotton wicks as described by Coppedge *et al.* [33] and placed in the front portion of the trap. Flies entered the WOT through a small hole and, once captured, they could remain alive for a few hours or even days [40]. After field tests, WOT proved to be better or equal to the collapsible trap in NWS captures in all collecting days and sites [40,41]. WOT also caught a lower number of *C. macellaria *than the collapsible trap [40,41]. 

Peterson II [42] evaluated the influence of trap color and height above ground on the capture of screwworms in WOT. More NWS flies were attracted to yellow and standard (yellow pail) WOT and to traps hung 0.4 m above ground than at the usual height of 1.5 m.

The WOT baited with *Swormlure*-4 was rapidly incorporated into the Screwworm Eradication Program as a replacement for liver baited traps and served as the standard detection system. For many years, this system was used extensively by the United States Department of Agriculture (USDA) to determine the first appearance of NWS in new areas and to serve as a quality control (distribution and survival parameters) for the released sterile insects. 

Coppedge *et al.* [43] reported that as few as five traps in a county were about equal to rancher reports in detecting native NWS. The standard procedure used by eradication officials became to operate approximately 20 WOTs in critical counties at fixed locations, with additional traps (on a short-term basis) being operated in areas where cases had been reported [35], coupled with case reports. 

In certain situations (such as on ships, in stables and in other smaller areas), sticky traps or electrocuter grid traps can be much more efficient than WOT [39,44,45] and may be used to supplement a grid of WOTs baited with *Swormlure*. Solar power can keep the batteries of portable, electrocuter grid traps charged, but this alternative is more costly [44].

Wild NWS populations are not considered to exceed 200 female adults/km^2^ [46]. Adults prefer well-wooded riverine areas, moist well-shaded sites, dense forests, and areas close to high concentration of hosts [47,48,49,50]. Some factors may reduce the effectiveness of WOT baited with *Swormlure*-4 in tropical dense forests [38], which may include reduced size of attractant plume, saturation of the woods with the attractant, or the settling of the plume to the ground as a result of higher humidity and low wind movement in the woods [51]. The exact positioning of adult traps, therefore, depends on geographical, ecotone, and host density considerations, but they should preferably be placed in isolated trees in pastures or in the interface between paddock and forest [52,53]. Furthermore, the lack of competing natural resources available during dry seasons usually enhances the efficacy of attractants to screwworms [53,54]. 

## 4. Methods of Control

Following IPM principles [15], a set of strategic techniques can be applied to suppress or eradicate NWS populations. With adequate animal management, some types of injuries can almost disappear in livestock, resulting in lower frequency of myiasis cases. The use of larvicides to treat infestations and to protect animals is essential for effective larval control. Toxic bait systems, such as the Screwworm Adult Suppression System (SWASS) [55], were developed to attract and kill adult individuals. Once a low density level of the pest is reached, sterile flies can be released to regulate or inhibit the reproductive success of the target population [56]. 

### 4.1. Reduction of Injuries in Livestock

The NWS requires soft tissue of living warm-blooded animals for larval development [1]. Usually, larvae invade the disrupted epidermis and aggregate in small cavities, but they also have the capability to strike the intact inner corner of the eye and the perineal region without obvious trauma or hemorrhage. Foot abscess and lesions with pus are other sources for myiasis.

Parish [8] investigated the factors predisposing animals to NWS infestation in eight classes of livestock. The author reported 6,148 myiasis cases from 1936 to 1940 in Texas. From the total number of infestations, 41% of them occurred in sheep, 22% in goats, 21.5% in lambs, 6.5% in calves and 4.5% in cattle. Of the 43 studied factors, the wound caused by needlegrass was responsible for 1,178 cases (or 35% of myiasis cases in lambs and 27% in sheep). Of the eight types of wounds that may be easily eliminated by adequate sanitary management, husbandry practices were by far the most important, representing 63% of the infestations in calves and 8% in cattle. The most frequently infested man-made wounds were shear cuts, which were responsible for 48% of the cases in goats and 73% in kids. 

Krafsur & Lindquist [57] observed similar distribution of myiasis cases in Libya, where 74% of NWS cases were confirmed in sheep, 16% in cattle, 4% in goats, 4% in equines, and 2% in camels. In Venezuela, however, dogs represent the second most important host [11].

Changes in management can virtually eliminate several predisposing causes of screwworm infestations [8] from the previous mentioned hosts. Injuries caused by needlegrass, for instance, can be controlled by proper grazing (especially during the spring growing season) and shearing all classes of sheep on the face and legs. 

The incidence of myiasis resulting from breeding and birth (irritation, mucosal abrasion, and secretions in the vulvar region are attractive to flies) can be reduced by limiting them to periods of the year when NWS is less prevalent [8]. 

Some man-made wounds can be prevented by following proper practices and more careful handling of livestock. Shear cuts can be greatly reduced by applying suitable wound protectors and proper timing of shearing (*i.e.*, times of the year with low prevalence of NWS). The same may be applied for other practices such as branding, castrating, dehorning, docking, earmarking, and registration tagging [8]. Another cultural method to reduce unnecessary wounds is to remove thorny trees and barbed wire from animal paddocks. 

Ticks, horn flies and stable flies are also responsible for inciting screwworm attacks [8]. They feed on various parts of the body, causing vulnerable wounds. Cultural, biological, and chemical tactics can be used to control them [58,59]. 

Some wounds predisposing myiasis cannot be extensively controlled, such as stone bruises, snake bites, fighting, hooking, and udder abnormalities, among others. Dehorning cattle when they are calves may be helpful. Females that present distorted udders should be used only for wool or mohair (in the case of sheep and goats) or beef (in the case of cattle), being marketed as soon as their condition warrants [8]. 

More recently, many farmers and livestock companies have adopted stricter animal welfare standards in order to meet requirements of customers and consumers worldwide. Welfare practices dictate that farm animals must be respected by producers, transporters, and operators during breeding, handling and slaughter, besides being provided with an appropriate environment. Fear, discomfort, stress, hunger, thirst, excess of heat or cold, injuries, and diseases must be avoided at every single step [60]. Ultimately, well bred and healthier animals with reduced therapeutic interventions may yield best performances and high quality products. 

### 4.2. Larvicides

Protection from screwworm infestation and parasite control of livestock can be achieved by spraying, dipping, or injecting larvicidal insecticides. The first success administration of an insecticide to control a bloodsucking insect was attained by Knipling [61], who rendered manure toxic to horn fly larvae by feeding phenothiazine to cattle. Bushland [62] mixed test materials in mason jars (“jar method”) containing larval diet (ground beef, citrated blood, water and formalin) and found that benzene derivatives could be useful in treating infested wounds. Bushland [63] used the same method to test 551 organic compounds (nitro compounds, halogen derivatives, phenols, compounds containing methoxyl or ethoxyl radicals, primary amines, azo compounds, hydrocarbons, and quinoline derivatives) compared to phenothiazine and rotenone for NWS toxicity. In 1943, Lindquist *et al.* [64] found that rabbits tolerated DDT and pyrethrum extract given orally in sufficient amount to render the blood toxic to bed bugs. Knipling *et al. *[65] found that lindane and 2-pivalyl-1,3–indandione given to rabbits orally, subcutaneously, intramuscularly, or intravenously rendered the animals toxic to body lice (*Pediculus humanus* L.) and mosquitoes (*Aedes aegypti *L.). These findings, made at the Orlando, Florida, laboratory of the USDA in conjunction with wartime research on the development of military insecticides, justified the beginning of a project on animal insecticides at the Kerrville, Texas, Livestock Insects Laboratory.

As testing against NWS larvae offered many advantages (*e.g.,* easy rearing, short life cycle, and diversity of hosts), *C. hominivorax* was selected as a screening insect [66]. In general, screwworms are more sensitive to screening materials than stable flies or ticks, since they are exposed continuously to the insecticides in blood, other fluids, and tissues (ticks, for instance, are very slow feeders and ingest only small amounts of blood containing various concentrations of the insecticide and its metabolites) [67].

In the screwworm testing program, a test insecticide was given to artificially infested guinea pigs or sheep. The researchers dropped consideration of a chemical if larvae continued to grow normally after hosts had been given a maximum dose. Residual effectiveness of a treatment was checked by periodically reinfesting a wound with newly hatched larvae [68]. Other techniques for observing effects on rate of larval growth, pupal mortality, and adult emergence, survival, and fertility were available when any special circumstance justified their use [68]. Since 1940, tests with hundreds of candidate compounds and small-scale field tests with promising larvicides continued to be conducted [69,70,71,72].

In 1958, the Animal Disease Eradication Division of the USDA began a campaign to eradicate the NWS from Florida and other southeastern States, and 13 livestock-inspection stations were established along the Mississippi and Pearl rivers to prevent any shipment of screwworm infested animal into the eradication zone. All livestock entering the eradication zone were unloaded, thoroughly sprayed with coumaphos, and wounds were smeared with EQ-335 (based on 3% lindane) wound dressing or Smear-62 (based on 35% benzol and 10% turkey red oil). Other commonly used smears were based on 5% toxaphene, 2% coumaphos or 2% dioxathion [73]. 

Spraying livestock with insecticides or to smear wounds with insecticides formulated in viscous carriers became widely recommended methods of treatment and are still very used by farmers especially in developing countries. Organophosphorus compounds (*e.g*., coumaphos, chlorfenvinphos, diclorvos, diazinon, dichlofenthion, fenchlorvos, fenthionethyl, and trichlorfon, among others) are commonly applied as aerosol sprays (in which marker dyes and bacteriostats are also included) or as dusts that are puffed into the wound from plastic squeeze bottles. Individual treatment sachets (*e.g*., 5 g of 5% coumaphos wettable powder) can also be either sprinkled directly on to a wound or brushed into the wound as a paste after mixing with cooking oil [74]. Wrich [75] showed that coumaphos was more effective than ruelene or runnel dusts in killing and preventing screwworm reinfestation for 48 h. Drummond *et al.* [76] evaluated 19 insecticides sprayed onto infested cattle, and most of the organophosphate compounds presented complete control of 1-2 day old larvae.

Chlorinated hydrocarbons (*e.g.*, aldrin, dieldrin, DDT, lindane) are effective against NWS [68], but their use became impractical because of excess of residues in fat [77], and being banned from many markets. Pyrethroids (*e.g.*, cypermethrin, cyhalothrin, deltamethrin) have potential for control of NWS myiasis and are currently being applied in combination with organophosphorus compounds [78]. Spinosad, a formulation based on a compound found in *Saccharopolyspora spinosa*, was effective in treating and preventing NWS myiasis when applied as an aerosol spray [79].

Smears and sprays reduce drastically the infestation at the time of application, but the protective period usually lasts only a week or less, and animals have to be regrouped and retreated adequately to prevent reinfestation [77]. Under extensive grazing conditions, where animals are not examined at short intervals, repeating treatments is expensive and not practical. 

Although the investigations had started in 1943, it was not until 1956 that ronnel, the first practical systemic insecticide, was announced [77]. The close cooperation during the following decades among the USDA, chemical companies, and team work of entomologists, veterinarians, and chemists, accelerated the development of novel systemic insecticides. The development of injectable systemic insecticides with high efficiency and prolonged prophylactic effect represented a major breakthrough to livestock industry.

Controlled-release formulations and devices can provide season-long protection for livestock with a single application of the insecticide. Gladney [73] showed that tags with stirofos or dichlorvos could provide 97%–100% simultaneous control of the Gulf Coast tick (*Amblyomma maculatum *Kock) and *C.hominivorax* for at least 7 weeks after one application of a single ear tag. Ahrens *et al.* [80] verified that 15% stirofos ear tags were more effective than ear bands and neck bands for 14 weeks, whilst petroleum base aerosols were effective for only 4 weeks against Gulf Coast ticks and screwworms in Texas. 

A single subcutaneous injection of ivermectin (200 µg/kg), a macrocyclic lactone produced by *Streptomyces avermitilis*, has been effective against NWS in preventing navel strike of newborn calves and scrotal strike of castrated calves [81]. Studies involving ivermectin and abamectin activity have been reported in Argentina and Brazil under field conditions when the treatments were administered to newborn calves or at castration [82,83]. Anziani *et al.* [84] reported that subcutaneous injection of abamectin provided good, but not 100% prevention of post-castration myiasis by NWS.

There have also been an increasing number of publications reporting that a subcutaneous injection of doramectin (200 µg/kg) can be up to 100% effective as a NWS prophylactic, preventing infestation of artificial wounds, umbilical or castration wounds of calves, and infestation of post-parturient cows for up to 12–15 days after application [85,86,87]. Compared to doramectin, the residual action of ivermectin appears to be shorter [88]. Although these endectocide molecules are similar, differences in doramectin formulation and probably a slower metabolic rate may contribute to a higher plasma availability compared to abamectin or ivermectin [87,89].

Effectiveness also depends on factors such as cattle breed and degree of fly strike. In general, the larvicidal activity of these endectocides remains as one of the most effective approaches to control screwworms, as routine management practices (branding, calving, castration, *etc.*) are allowed to be scheduled to maximize productivity rather than being limited to times of the year when the NWS is less prevalent.

There are indications that fipronil, a phenyl-pyrazole, can be effective as larvicide. Silva *et al.* [90] reported that the curative efficacy of fipronil 1% pour on in castration wounds was 100%, with prophylactic effect in more than 95% of the treated Zebu crossbred bulls over the critical 10 day post-castration period (when most ovipositions occurred). Nitenpyram, a neonicotinoid used for flea control in domestic animals, has showed 100% efficacy on the treatment of NWS myiasis in naturally infested dogs [91].

Environmentally friendly insecticides such as insect growth regulators can also be effective against NWS. Active juvenile hormone analogues, methoprene and R-20458, inhibited the emergence of *C.hominivorax* when applied topically to larvae and when incorporated within the larval diet [92]. When NWS female adults were fed on diet containing diflubenzuron, egg hatch was greatly reduced [93]. A single topical application of dicyclanil in castrated calves also provided good protection (≥95%) against NWS myiasis [94].

Nevertheless, the intensive use of chemical control since the 1960s has led to the selection of resistant populations in some countries. Knipling [95] proved that NWS larvae could become resistant to phenothiazine through generations. Rawlins *et al.* [96] conducted a survey of resistance to insecticides among NWS populations from various geographical regions and found that the relative resistance of a Jamaican strain was 78 fold to coumaphos. The same authors attempted to select resistant strains from adults and observed, after 5-6 generations, maximum tolerance of 6.5 fold to dieldrin and coumaphos. Investigations of genes and mutations involved in insecticide resistance have also identified populations resistant to organophosphorus compounds and pyrethroids in South America [78,97]. 

### 4.3. Screwworm Adult Suppression Systems

The development of effective chemical attractants for the NWS [33,38] raised the possibility of incorporating an attractant into a toxic bait system. In the 1970s, scientists of the USDA began to work on suppression units for adult screwworms which would consist of three parts: a long range attractant, a feeding bait formulation, and an insecticide (probably combined with the feeding bait). 

The initial step in the development of the toxic bait system was to select an appropriate feeding attractant. Tannahill [98] showed that a simple mixture of dried blood, granulated sugar and water (1:2.5:2, wt/wt/wt) proved to be very attractive and last for several days. However, it was dissolved by rains and heavy dews, a problem that was overcome by replacing water with Elmer’s glue (the new ratio for sugar, dried blood, and glue was 1:2.5:4, wt/wt/wt) [55]. The second step was to find an insecticide and dosage that could give a fast knock-down of the flies feeding on the mixture with it. Coppedge *et al.* [55] demonstrated that a 2% dichlorvos bait combination was not repellent to attracted flies and was effective in killing them. 

The final step consisted in evaluating devices which would offer the toxicant formulation. According to Coppedge *et al.* [55], the most effective device was a cardboard cylinder (5.1 by 7.5 cm). The toxic bait mixture (5 g of a combination of 2% dichlorvos, 14% dried blood, 32% granulated sugar, and 52% glue by weight) was smeared in a band *ca.* 2.5 cm lengthwise inside the cylinder, and a 2.5 or 4 cm long piece of nº 2 dental roll containing 2 mL of *Swormlure-2* was placed *ca.* half way down the cylinder [55]. The flies were attracted to the *Swormlure-2* and then fed on the toxicant bait. The majority of flies usually died within a 15 cm radius of the cylinder [99]. This was the first Screwworm Adult Suppression System (SWASS) unit developed [55]. 

When these units were evaluated in the area of the sterile insect mass-rearing facility (in which vicinity thousands of sterile flies were available weekly), most units gave kills of sterile flies in excess of 100/h/SWASS unit [55]. In a preliminary field test conducted on a ranch located 97 km north of the mass-rearing facility, Texas, wild screwworms were attracted and killed, with 75%–80% of the flies killed being females (*ca.* 1 male: 5 females ratio) [55].

The first opportunity to test the new suppression system in large-scale and against an entire screwworm population occurred in Curaçao, a relatively small (460 km^2^) Caribbean island in the Netherlands Antilles, 6.5 km from Venezuela coast [100]. From 1954 until December 1975, this island remained free of NWS. However, in December 1975, a screwworm infestation was reported from the island (reinfestation was probably due to shipment of livestock from Venezuela or Colombia), and by June 1976, *C. hominivorax* had become firmly reestablished, presenting a serious human and animal health problem [100]. At the invitation of the local officials, an eradication experiment was started and consisted of three phases: (1) adult survey, case incidence reporting system, and re-education of public; (2) distribution of SWASS units; (3) release of sterile insects. In August 1976, WOTs were distributed over the island (*ca.* 100 traps, each baited with 60 mL of *Swormlure-2*). The preliminary trapping and data collection provided a reliable base-line data for about 6 months prior to the release of the suppression units [100]. The SWASS units [55] were dropped weekly (10–20 km^2^) over the rural part of the island by airplane by using a system developed by Goodenough *et al.* [101] and dispersed by ground (10–20 km^2^) in the city of Willemstadt for a period of 15 weeks from February 26 to May 13, 1977 [100]. After this period, collection of adults in survey traps indicated 65%–85% suppression, and reported larval incidence declined 30-70% [100]. No reports of injury to domestic or wild animals, birds, people, *etc.* were reported. In mid-May, the distribution of SWASS units was discontinued, and releases of sterile flies (1,575 sterile flies/km^2^/wk) were initiated. The low residual NWS population was eradicated in 3-4 weeks, and Curaçao was declared screwworm free on October 25, 1977 [100]. The success of this experiment confirmed Knipling’s estimates that, considering a normal rate of population increase, a daily mortality of 10% of the females before they first oviposit would result in an 87.5% reduction in the target population after two generations [55]. 

Although effective, the original SWASS units were bulky and not very amenable to mass production. These constraints were of major importance as SWASS was being considered to be converted from an experimental system to an operational part of the eradication campaigns. Efforts were, therefore, made to develop new formulations. In 1980, Coppedge *et al.* [102] reported a new pelletized formulation.

The SWASS pellets (3–4 g; 1.27 cm in diameter and 2.5 cm long) were prepared from 30.5% sugar, 30.5% dried blood, 7.5% corn cob grits, 2% dichlorvos, and 29.5% of a hardened wax preparation of *Swormlure-2* (82% *Swormlure-2*, 18% wax) [102]. When stored at 1.1 °C in aluminum-lined bags, the pellets had a storage life of about 8 weeks (25% of the dichlorvos was lost and 10% of the *Swormlure*) (at room temperature, the storage life was only 7 days) [35]. 

The new SWASS units were field tested for the first time on a large-scale basis against a non-isolated NWS population in a 518 km^2^ area of the Davis Mountains, Jeff Davis county, Texas, during 1978 [102]. An outbreak had occurred in the area and was beyond control by the conventional release of sterile flies [35]. The SWASS pellets were applied aerially (58 pellets/km^2^) twice weekly (at 2 to 5 day intervals) on lanes 1.6 km apart, with the second application flown between these lanes [102]. The pellets reduced the populations of *C. hominivorax *and *C. macellaria* by *ca.* 72%–95% and 90%, respectively, and proved to be about three times more effective than the original SWASS cylinder during a 7 day period (the cylinder was effective during the first 24 h, while the pellet was highly attractive for 3 days) [102]. 

Tannahill *et al.* [103] performed a large scale evaluation of SWASS pellets on the Pacific Coast of Mexico. The pellets were distributed by airplane (58 pellets/km^2^/application) twice weekly over an area of 2,625 km^2^ in a period of 3 weeks. After the 6 applications, the number of NWS adults captured in traps was reduced 72% and the number of egg masses collected was reduced 81%, proving the effectiveness of the new system even in a tropical rainy region. 

With the success of SWASS evidenced in semi-arid and tropical areas, special-use registration for this suppression system in Texas, Arizona, and New Mexico was accomplished in March 1979, and the pellets started being used in outbreaks (which were defined as more than three cases within an area of 1,024 km^2^ or the capture of 0.5 wild flies/trap in a similar area) [35], areas where screwworm infestations persisted despite the release of sterile flies, or in areas previously untreated with sterile flies where adult surveys and animal infestations indicated very high populations [104]. 

The pelletized formulation of SWASS started being used operationally in the joint U.S.-Mexico Screwworm Eradication Program in the following years. The standard treatment became an air dispersal of pellets over infested areas at the rate of 60 pellets/km^2^ twice per week on a 0.8 km swath width [104]. It was standardized that at least the entire area within 16 km on all sites of reported cases and captured wild flies should be treated during an eradication campaign [35]. Until the early 1980s, SWASS was used successfully over 50 areas of Arizona, Texas, New Mexico, and Mexico, and in most cases no more than four applications were necessary to effectively suppress the target populations [35]. 

Dichlorvos could also be replaced by alternative insecticides that would not be repellent and could remain effective for at least as long as the *Swormlure* was attractive. Peterson II *et al.* [105] reported that trichlorfon should be considered as an alternative insecticide for use in SWASS pellets, since it was longer lived (93.8% trichlorfon remained in the pellet after 7 days compared with only 14.8% dichlorvos), as effective as pellets containing dichlorvos (trichlorfon pellets gave 50%–83% of field population suppression), less needed (1% as compared with 2% dichlorvos), and slightly less toxic to mammals than dichlorvos. 

Mackley & Brown [104] compared the efficacy of the standard pellet (12.7 mm in diameter) and two smaller-diameter sizes (9.5 and 6.4 mm). The standard pellet attracted more adults per pellet than did the 9.5 and 6.4 mm pellets. More *Swormlure-4* evaporated and more dichlorvos remained as pellet size increased. In many cases, the amounts of dichlorvos fell below acceptable levels after only 48 h of exposure, but the standard and 9.5 mm pellets contained similar percentages of the insecticide after exposure up to 4 days. Exposed pellets lost more *Swormlure-4* and dichlorvos. The 6.4 mm pellet was considered ineffective and, therefore, unsuitable for application in the eradication campaign. 

Considering the environmental impact of SWASS applications, Peterson II *et al.* [99] determined which non-target organisms were attracted to the pellets at 391 sites in Texas, New Mexico, Arizona, and Mexico. A total of 4,640 insects of 168 species were collected near SWASS pellet sites. Diptera comprised 93.5% of the insects collected, and 88.5% of these were Calliphoridae, Sarcophagidae, Anthomyiidae, and Muscidae. *C. macellaria* represented 48.2% of the total number of specimens collected. None of the species killed were noted as important beneficial species, and immigration from outside the SWASS treated area would quickly repopulate it if any non-target species had been drastically reduced [35]. Mammals caught in traps baited with 1–2 pellets included mostly rats, but the pellets were not eaten, just chewed or moved. Domestic animals (dogs, cats, sheep, goats, cows, hogs, and chickens) had little or no interest in the pellets. On the average, 10% of all pellets were degraded within one week, and long term impact on non-target populations appeared to be not relevant. 

A constraint for the SWASS pellets was that they could not be dropped from air in certain areas, such as near lakes and other major accumulations of water, rivers, certain areas supporting endangered wildlife, and where the system was not registered for use. Coppedge *et al.* [106] then decided to explore a bait station approach in order to find a long-lasting toxic bait system for ground distribution. The developed bait station consisted of a paper cone (15 cm diameter) covered on top with 50 g of feeding bait-toxicant mixture (dried blood, sugar, glue, and 4% dichlorvos) [55] suspended over a 40 g cup of solidified *Swormlure-2 *(9.6 g of wax for 30 mL of *Swormlure*). In field tests, bait stations were equal or superior in effectiveness to WOTs for killing or capturing NWS for 45 to 60 days. Their results also indicated that a target population could be reduced at least 25 to 30% with bait stations at a density of 1 trap/2.6 km^2^, and that a random distribution of the baits over an area could be more effective than clumping them around watering sites. 

From its first design until the bait station approach, SWASS proved to be highly effective in high population situations [35]. Thus, SWASS provided a method to rapidly reduce large NWS populations, making them more manageable for complete suppression or eradication. 

### 4.4. Sterile Insects

The Sterile Insect Technique (SIT) can be defined as “a method of pest control that uses area-wide inundative releases of sexually sterile insects to reduce fertility of a field population of the same species” [107]. Before its application for controlling any given pest, a number of assumptions must be considered: (1) availability of mass rearing methods of the insect; (2) the mating behavior of the insect must not be adversely affected by the sterilization procedure; (3) the released sterile insects must present adequate dispersion in the field; (4) the female of the target species must normally mate only once; (5) the population density of the insect must be low [56]. 

The mass rearing of the target insect, in sufficient number and of adequate quality, is one of the main requirements for the implementation of the technique. In the 1930s, Melvin & Bushland [108] developed an artificial diet and procedures to colonize the NWS. In the following decades, other rearing systems for NWS larvae based on hydroponic diet [109], gelled diets [110,111,112], and cellulose fiber-based diet [113] were developed. Studies on adult diet [114,115] were equally important to make insect production timely and cost effective (*e.g.*, Chaudhury *et al.* [116] developed a meatless adult diet which represented savings of about US$ 100,000/year for the NWS mass rearing facility located in Mexico). The complete procedures and steps for mass production of sterile NWS flies for area-wide eradication campaigns in the USA were described by Smith [117] and Baumhover *et al.* [4].

Sterility in screwworms can be induced by chemosterilants [118,119] when added to diets, sprayed over the insect or even in attractant baits in the field. However, most of the chemosterilants are mutagenic, what leads to human health and environmental issues, besides the fact that insects can develop resistance [96,97]. Thus, for the past five decades, exposure to ionizing radiation has been the most used means of inducing sterility in mass-reared screwworms for AW-IPM campaigns that integrate the SIT [120].

Muller [121] demonstrated that sterilization could be induced in irradiated *Drosophila melanogaster*. Some radiations can make insects reproductively sterile by specifically causing germ-cell chromosome fragmentation (dominant lethal mutations, translocations, and other chromosomal aberrations) that leads to the production of imbalanced gametes and, subsequently, the inhibition of mitosis and death of fertilized eggs and embryos [120]. In 1946, Lindquist called Knipling’s attention to the fact that Muller had reported a means of sterilizing mass-reared insects. 

During 1950, Bushland & Hopkins [122] conducted the first experiments to irradiate NWS using a deep-therapy machine at the X-ray Therapy Section of the Brooke Army Hospital, Fort Sam Houston, San Antonio, Texas. They observed that females mated only once, and when *ca.* 50 Gy of X-rays were applied to pupae during their last two days before emergence, the adults that emerged were sterile but could compete equally with non-irradiated flies. The development of gamma ray sources by the Atomic Energy Commission [123] offered the possibility of irradiating a larger number of insects per batch, as the penetration and dose rate achieved by most X-ray machines at that time were much lower.

Bushland & Hopkins [124] then compared gamma and X-rays, and found no difference between them. They also concluded that no dose below 48.7 Gy was sufficient to induce 100% sterility (lots with males and females) and that irradiating pupae 5 days or older would be the best procedure. Baumhover [125] investigated the influence of aeration during gamma irradiation and observed that when pupae were irradiated in a carbon dioxide atmosphere, a higher dose (*ca.* 108 Gy) was required to sterilize the females. Two decades later, Crystal [126] re-examined the sterilization doses trying to find an optimal balance between dose and insect age, and realized that when pupae were irradiated 72 h before the adult emergence, the sterility of males irradiated at 20 Gy or more was higher than 95%, and females were 100% sterile at 40 Gy. 

Irradiation presented many advantages over other sterilization methods as insects could be irradiated inside packaging materials, the sterile insects could be released immediately after irradiation, residues that could be harmful to humans or the environment were nonexistence, and temperature rise during the process is usually insignificant [120]. The induction of radioactivity in irradiated material for SIT operations is prevented by ensuring that the energy applied is below 5 million electron volts (MeV) for photons (gamma or X-rays) or 10 MeV for electrons [120,127]. 

The first published field evaluation of sterile screwworm flies was performed during 1951-1953 at Sanibel Island (47 km^2^), 4 km off the coast of Florida. The results corroborated the previous laboratory studies, and just after 8 weeks of releases (*ca.* 39 sterile males/km^2^/wk), 100% of the egg masses collected from wounded sentinel goats were sterile. Nevertheless, eradication was not achieved because wild fertile flies continued reinfesting the island from the mainland [24]. 

To prove the SIT effectiveness once for all, an eradication trial was initiated in Curaçao Island in 1954. The NWS flies were reared in the USDA Entomology Research Division Laboratory at Orlando, Florida, and irradiated pupae were packaged in paper bags, air shipped to Curaçao, and released by air twice a week. The sterile males started being released (*ca.* 155 sterile males/km^2^/wk) on August 1954 and eradication was accomplished within just 14 weeks [24]. 

The successful eradication of the NWS from Curaçao increased interest in the SIT for eradication of this pest from southeastern USA. During 1955, additional rearing facilities were constructed near Bithlo, Florida, with the initial goal of producing 1 million flies/wk (or 6 times the Curaçao production). This goal was rapidly achieved by expanding operations based on procedural modifications adopted for Curaçao [128]. In the spring and summer of 1957, livestock owners experienced the worst losses from NWS since its first detection in the Southeast in 1933. In the same summer, the Entomology Research Division of the USDA, in cooperation with officials of the Florida Livestock Board and the Animal Disease Eradication Division of the USDA (who were to administer the state-federal campaign in southeastern States) planned a pilot field test with sterile screwworms. Releases in a field test (772.5 km^2^) southeast of Orlando were begun on May 1957, at the rate of 193 sterile males/km^2^. Despite the initial outbreak proportions of the infestation, sterility of egg masses reached 70% during July. The test was considered successful, and realistic planning for eradication was initiated [128]. 

The Florida State legislature then appropriated US$ 3 million to share the cost of a joint federal campaign, which led to the implementation of the eradication effort in the southeastern USA. In early 1958, a new mass rearing plant was planned to operate in a former US Air Force base near Sebring, Florida. The campaign got off a good start due to the winter of 1957–1958, which was one of the coldest in the history of Florida. To take advantage of this severe winter, sterile fly production at the Bithlo facility was increased from 2 million/wk to 14 million/wk. The new facility near Sebring was officially opened on July 1958, and within three months it was producing over 50 million/wk [128]. During 1958, sterile flies were released at the rate of *ca.* 78 flies/km^2^/wk throughout peninsular Florida. During the campaign, special releases were made in several areas in which screwworms persisted and, because sporadic outbreaks of wild flies occurred, sterile flies were released as far north as southern Georgia to maintain a 63 km barrier. Releases were discontinued on 3,861 km^2^ in southern Florida in late July 1959 and in entire Southeast on November 1959. After this two-year campaign (1957–1959), the southeastern USA was declared NWS free. Campaign cost was US$ 10 million, or half the annual loss to livestock owners from NWS infestations [128]. 

Livestock producers in Southwest watched that eradication campaign with much interest, and some representatives of the livestock industry in Texas visited the Sebring plant. Soon afterwards, the Southwest Animal Health Research Foundation (SWAHRF) was formed to obtain support for an eradication campaign in the southwestern USA [129], and from 1960 to 1962, SWAHRF struggled to raise funds. Livestock owners gave US$ 3 million to match US$ 3 million from the State of Texas. An additional US$ 6 million in federal funds brought the total to the US$ 12 million required for a three-year campaign. In phase I, eradication from the overwintering area in Texas would be attempted. If successful, phase II would test the feasibility of a sterile-fly barrier in Mexico [128]. 

In February 1962, the southwestern campaign was inaugurated in a state of urgency to take advantage of the weather that was unfavorable to the NWS (as occurred in the Southeast in 1958), and the Animal Disease Eradication Division of the USDA-ARS expanded a rearing facility in Kerrville, Texas. From February to June 1962, SWAHRF constructed a new mass rearing facility at Mission, Texas, with a capacity to produce 200 million sterile flies per week. Isolated infestations (in Texas, Louisiana, Arkansas, Oklahoma, and New Mexico) and along major watercourses in and beyond the primary grid zone were treated at *ca.* 770 sterile flies per linear km. During the winter 1963–1964, releases were extended 125 km into strategic areas throughout northern Mexico, and only 233 cases were recorded in Texas during 1964. In 1965, Arizona and California joined the campaign. After 1963, with the southwestern campaign in operation, none of the southeastern States reported a single case of NWS infestation. In 1966, the entire USA was declared screwworm free [128]. The southwestern campaign, costing US$ 5 million/year, saved livestock owners in Southwest about US$ 50–100 million each year, and additional savings were enjoyed by ranchers in northern Mexico [4]. 

Due to continued outbreaks in USA between 1966 and 1972, and interest of Mexican livestock producers in extending the eradication campaign into Mexico, it was decided to move the sterile fly biological barrier south to the Isthmus of Tehuantepec in Mexico (a 190 km width as compared to 2,400 km at the US-Mexico border) [129]. 

An agreement was signed on August 1972, creating the *Comisión México-Estados Unidos para la Erradicación del Gusano Barrenador del Ganado *(COMEXA). The goals of the COMEXA were to construct a new mass rearing facility and to eradicate NWS southwards [129]. The campaign got underway in 1972, using sterile flies from the plant at Mission, Texas. The COMEXA sterile fly mass production facility in Tuxtla Gutierrez, Chiapas, was inaugurated in August 1976 and had the capacity to produce 500 million sterile flies per week [129]. The campaign in Mexico continued using sterile flies from both facilities until the Mission plant was closed in 1982. The goal of eradicating NWS to the Isthmus of Tehuantepec was achieved in 1984. However, further studies demonstrated that a permanent barrier extending from the Panama Canal to the border with Colombia would be more advantageous, requiring only 40 million sterile flies/wk instead of 150 million/wk needed in the Isthmus of Tehuantepec [129].

Following indications of interest from practically all the Central American countries and Panama, a plan was traced in 1985 to extend the eradication campaign through Central America and establish the biological barrier in Panama. After the enlargement of the campaign in Mexico to cover the entire country, in February 1991 Mexico was declared screwworm free [129]. The COMEXA then signed an agreement with Guatemala government in December 1986. At the peak of aerial dispersal, *ca.* 115 million sterile flies/wk were dispersed to cover the entire Guatemala. Dispersals ceased at the end of December 1993, and an official declaration of freedom from NWS was held on May 1994 [129].

Dispersal of sterile flies began over Belize on August 1989. At the peak of dispersal, 24 million sterile flies/wk were released. Belize was officially declared NWS free on May 1994 [129]. 

Aerial dispersal of sterile flies over El Salvador started in October 1991. An average of 24 million sterile flies/wk were dispersed. In June 1995, El Slvador was declared screwworm free. Also in late 1991, sterile fly dispersal over Honduras was begun. By June 1993, up to 120 million sterile flies/wk were dispersed. In August 1996, Honduras was free from NWS [129].

In July 1993, releases of sterile flies were initiated in Nicaragua. Approximately 120 million sterile flies/wk were required to disperse over the entire country. The last myiasis case by NWS in Nicaragua was reported in June 1997. Flights operating out of Nicaragua were also used to disperse sterile flies in Costa Rica on early 1996. About 60 million sterile flies/wk were needed to cover Costa Rica, and the last reported case occurred in March 1999 [129].

Dispersal of sterile flies over Panama began in July 1998, also using over-flights out of Nicaragua. In October 1998, 50% of the country was dispersed from a new dispersal center located at Tocumen International Airport near Panama City. Approximately 80 million sterile flies/wk were needed to cover the entire country at the programmed rate of 3,000 sterile flies/km^2^ dispersed in 3.2 km swath widths [129]. Panama was declared screwworm free on July 2006, and a new mass rearing facility was inaugurated by the *Comisión Panamá-Estados Unidos para la Erradicación y Prevención del Gusano Barredor del Ganado* (COPEG) at Pacora. 

Currently, only the mass rearing facility at Pacora is capable of providing millions of sterile screwworm flies per week, and the buffer zone of 30,000 km^2^ at the Darien Gap is being maintained by the weekly release of 25–50 million sterile males [129,130]. The mass rearing facility in Tuxtla Gutierrez was closed with the dissolution of the COMEXA in September 2012 due primarily to economic reasons. 

Although the production and release costs of sterile NWS flies are not low (US$ 1.0/1000 insects and US$ 0.4/1000 insects, respectively), the costs/km^2^/year of the SIT operation are the lowest for NWS (US$ 182.00/km^2^/year) when compared to other pests target of AW-IPM programs that release sterile insects, such as the Mediterranean fruit fly (US$ 4,160.00/km^2^/year) [131]. 

The permanent sterile fly biological barrier between Panama and Colombia continues to protect the NWS free countries from reinvasion from South America, and the 50-year AW-IPM campaign ultimately resulted in estimated annual producer benefits in the USA, Mexico, and Central America of greater than one billion dollars [9].

The NWS was historically restricted to the Americas, but in March 1988, the NWS was discovered in Tripoli, Libya, infesting domestic animals, and during 1990 an explosive outbreak of screwworms occurred in a region of 26,500 km^2^ in northwestern Libya [57]. Shipment of infested livestock from South America was suspected to be the origin of the infestation. An eradication campaign, largely using regular inspection of animals and sterile insects, began in December 1990, coinciding with the onset of seasonally cool weather. The main agencies involved were the International Atomic Energy Agency (IAEA), the Food and Agriculture Organization of the United Nations (FAO) and the Libyan government. In May 1991, releases of 300–500 sterile males/km^2^ were made over 40,000 km^2^ and this continued until October 1991. From January to August 1991, almost 17 million animals were inspected at quarantine stations and by 70 teams of mobile surveillance teams. The last confirmed screwworm infestation in Libya was in April 1991 [57]. 

Efforts and isolated projects to suppress NWS populations continue in the Caribbean and South America. In 1996, an eradication campaign began in Jamaica, but it suffered many constraints and eventually failed [132]. Between January and May 2009, a pilot-test was performed at the Brazil-Uruguay border (a test area of 4,662 km^2^) with sterile flies imported from COMEXA and, after just 13 weeks of air releasing *ca.* 1,545 sterile flies/km^2^, 21.5% of the egg masses collected were sterile [133]. 

## 5. Conclusions

The New World Screwworm (NWS), *Cochliomyia hominivorax*, causes obligatory myiasis in warm-blooded animals and is a major pest of livestock in the Caribbean and South America. Before the adoption of control measures, AW-IPM campaigns against this pest require active detection and surveillance of NWS populations through inspection of animals and trapping systems.

The availability of different methods of control (*e.g.*, prevention procedures, larvicides, SWASS, and SIT) has strengthened previous AW-IPM campaigns against this pest. A campaign that depends solely on one component of control is much more vulnerable to failure since no backup method exists if the first system fails. Despite the appearance of lineages resistant to some insecticides, the chemical control and protection of livestock against screwworm strikes are still essential tools for improvement of animal health and management in tropical and sub-tropical areas.

Sterile flies were essential in eradicating the residual NWS populations in North of Africa and the Americas through to Colombia, and the SIT would be strategic in forthcoming projects against the NWS in developing countries. 

## References

[1] Hightower B.G., Spates Junior G.E., Garcia J.J. (1972). Growth and critical size at pupation for larvae of the screwworm developing in fresh wounds. J. Econ. Entomol..

[2] Thomas D.B., Mangan R.L. (1989). Oviposition and wound-visiting behaviour of the screwworm fly, *Cochliomyia hominivorax* (Diptera: Calliphoridae). Ann. Entomol. Soc. Am..

[3] Spradbery J.P. (1994). Screw-worm fly: A tale of two species. Agr. Zool. Rev. Bedfordshire.

[4] Baumhover A.H., Husman C.N., Graham A.J., Smith C.N. (1966). Screwworms. Insect colonization and mass production.

[5] Sereno J.R.B., Catto J.B., Sereno F.T.P.S. (1996). Use of Ivermectin to prevent navel myiasis in new born calves from Pantanal.

[6] IAEA (2008). Model Business Plan for a Sterile Insect Production Facility.

[7] James M.T. (1947). The Flies That Cause Myiasis in Man.

[8] Parish H.E. (1942). Factors predisposing animals to screwworm infestation in Texas. J. Econ. Entomol..

[9] Vargas-Teran M., Hofmann H.C., Tweddle N.E., Dyck V.A., Hendrichs J., Robinson A.S. (2005). 2005. Impact of screwworm eradication programmes using the sterile insect technique. Sterile Insect Technique: Principles and Practice in Area-wide Integrated Pest Management.

[10] Leão R.N.Q. (1997). Doenças Infecciosas e Parasitárias: Enfoque Amazônico.

[11] Coronado A., Kowalski A. (2009). Current status of the New World screwworm *Cochliomyia hominivorax* in Venezuela. Med. Vet. Entomol..

[12] Hall M., Wall R. (1995). Myiasis of human and domestic animals. Adv. Parasit..

[13] Skoda S.R. (2001). Kit for Detecting Flesh-eating Maggots. Science Update.

[14] Hendrichs J. (2001). To the reader. FAO/IAEA Insect Pest Control Newsletter.

[15] Kogan M. (1998). Integrated Pest Management: Historical Perspectives and Contemporary Developments. Annu. Rev. Entomol..

[16] Lindquist D., Tan K.H. (2000). Pest management strategies: Area-Wide and conventional. Area-wide Control of Fruit Flies and Other Insect Pests.

[17] FAO (1993). Manual for the Control of the Screwworm Fly Cochliomyia hominivorax (Coquerel). Guide for the Identification of Flies in the Genus Cochliomyia (Diptera: Calliphoridae).

[18] Dear J.P. (1985). A revision of New World *Crysomiini* (Diptera: Calliphoridae). Rev. Bras. Zool..

[19] De Leo´n D., Fox I. (1980). Canine minima myiasis in Puerto Rico—A case report. J Agric Univ PR..

[20] Spradbery J.P. (2002). A Manual for The Diagnosis of Screw-worm Fly.

[21] Lyra M.L., Hatadani L.M., Azeredo-Espin A.M.L., Klaczko L.B. (2010). Wing morphometry as a tool for correct identification of primary and secondary New World screwworm fly. Bull. Entomol. Res..

[22] Taylor D.B., Szalanski A.L., Peterson R.D. (1996). A polymerase chain reaction—Restriction fragment length polymorphism technique for identification of screwworms (Diptera: Calliphoridae). Med. Vet. Entomol..

[23] Litjens P., Lessinger A.C., Azeredo-Espin A.M.L. (2001). Characterization of screwworm flies *Cochliomyia hominivorax* and *Cochliomyia macellaria* by PCR-RFLP of mitochondrial DNA. Med. Vet. Entomol..

[24] BaumhoverA A.H., Graham A.J., Bitter B.A., Hopkins D.E., New W.D., Dudley F.H., Bushland R.C. (1955). Screw-worm control through release of sterilized flies. J. Econ. Entomol..

[25] Hammack L., Bromel M., Duh F.M., Gassner G. (1987). Reproductive factors affecting responses of the screwworm fly, *Cochliomyia hominivorax *(Diptera: Calliphoridae), to an attractant of bacterial origin. Ann. Entomol. Soc. Am..

[26] Parker F.D., Welch J.B. (1991). An alternative of sentinel sheep for collecting egg masses from wild females of the screwworm (Diptera: Calliphoridae). J. Econ. Entomol..

[27] Bishopp F.C. (1916). Fly traps and their operation. USDA Farmers Bull..

[28] Laake E.W., Cushing E.C. (1930). Fly trapping on the ranges of the southwest. J. Econ. Entomol..

[29] Bishopp F.C. (1937). Fly traps and their operation. USDA Farmers Bull..

[30] Grabbe R.R., Turner J.P. (1973). Screwworm attractants: isolation and identification of organic compounds from bacterially inoculated and incubated blood. Folia Entomol. Mex..

[31] DeVaney J.A., Eddy G.W., Ellis E.M., Harrington R. (1973). Attractancy of inoculated and incubated bovine blood fractions to screwworm flies (Diptera: Calliphoridae): Role of bacteria. J. Med. Entomol..

[32] Jones C.M., Oehler D.D., Snow J.W., Grabbe R.R. (1976). A chemical attractant for screwworm flies. J. Econ. Entomol..

[33] Coppedge J.R., Ahrens E., Goodenough J.L., Guillot F.S., Snow J.W. (1977). Field comparisons of liver and a new chemical mixture as attractants for the screwworm fly. Environ. Entomol..

[34] Broce A. B., Goodenough J.L., Snow J.W. (1979). Recovery of Screwworm Flies Released at Various Distances and Directions of the Attractant *Swormlure*-2. Environ. Entomol..

[35] Snow J.W., Coppedge J.R., Brace A.B., Goodenough J.L., Brown H.E. (1982). *Swormlure*: development and use in detection and suppression systems for adult screwworm (Diptera: Calliphoridae). Bull. Entomol. Soc. Am..

[36] Brown H.E., Mackley J.W. (1983). Changes in attractancy and chemical composition of the screwworm (Diptera: Calliphoridae) chemical attractant, *Swormlure*-2, under field condition. J. Econ. Entomol..

[37] Broce A.B., Davey R.B., Snow J.W. (1979). Plastic wicks as dispensers of the screwworm attractant, *Swormlure*-2. J. Econ. Entomol..

[38] Mackley J.W., Brown H.E. (1984). *Swormlure*-4: A new formulation of the *Swormlure*-2 mixture as an attractant for adult screwworms, *Cochliomya hominivorax* (Diptera: Calliphoridae). J. Econ. Entomol..

[39] Goodenough J.L., Snow J.W. (1977). Increased captures of adult screwworms and secondary screwworms in electrocuter grid traps. J. Econ. Entomol..

[40] Broce A.B., Goodenough J.L., Coppedge J.R. (1977). A wind oriented trap for screwworm flies. J. Econ. Entomol..

[41] Spencer J.P., Whitten C.J., Coppedge J.R., Snow J.W. (1980). Comparison of screwworm captures in liver and *Swormlure*-baited traps in a Tropical area of Southern Mexico. Southwest. Entomol..

[42] Peterson II R.D. (1982). Influence of Trap Color and Height above Ground on Captures of Screwworms. Southwest. Entomol..

[43] Coppedge J.R., Ahrens E.H., Snow J.W. (1978). *Swormlure*-2 baited traps for detection of native screwworm flies. J. Econ. Entomol..

[44] Goodenough J.L. (1979). Adult Screwworms: Comparison of Captures in Wind Oriented and Electrocutor Grid Traps. J. Econ. Entomol..

[45] Spradbery J.P. (1981). A new trap design for screw-worm fly studies. J. Aust. Entomol. Soc..

[46] Lindquist A.W. (1958). Entomological uses of radioisotopes. Radiation Biology and Medicine.

[47] Parman D.C. (1945). Effect of weather on *Cochliomyia americana* and a review of methods and economic applications of the study. J. Econ. Entomol..

[48] Brenner R.J. (1985). Distribution of screwworms (Diptera: Calliphoridae) relative to land use and topography in the humid tropics of Southern Mexico. Ann. Entomol. Soc. Am..

[49] Mangan R.L., Thomas D.B. Migration, habitat preference and reproductive patterns in natural populations of the screwworm fly, *Cochliomyia hominivorax* (Coquerel). Proceedings of 4th International Congress of Ecology.

[50] Mangan R.L., Thomas D.B. (1989). Habitat preferences and dispersal patterns in native screwworm flies (Diptera: Calliphoridae). Ann. Entomol. Soc. Am..

[51] Welch J.B. (1988). Effect of Trap Placement on Detection of *Cochliomyia hominivorax* (Diptera: Calliphoridae). J. Econ. Entomol..

[52] Parker F.D., Welch J.B. (1991). Influence of Attractants on Behavior of Screwworms (Diptera: Calliphoridae) in a Tropical Wet Forest in Costa Rica. J. Econ. Entomol..

[53] Parker F.D., Welch J.B., Matlock R.B. (1993). Influence of habitat, season, and attractant on adult behaviour of the screwworm (Diptera: Calliphoridae) in a tropical dry forest in Costa Rica. J. Econ. Entomol..

[54] Matlock R.B., Skoda S. (2009). Mark- recapture estimates of recruitment, survivorship and population growth rate for the screwworm fly, *Cochliomyia hominivorax*. Med. Vet. Entomol..

[55] Coppedge J.R., Broce A.B., Tannahill F.H., Goodenough J.L., Snow J.W., Crystal M.M. (1978). Development of a Bait System for Suppression of Adult Screwworms. J. Econ. Entomol..

[56] Knipling E.F. (1955). Possibilities of insect control or eradication through the use of sexually sterile males. J. Econ. Entomol..

[57] Krafsur E.S., Lindquist D.A. (1996). Did the sterile insect technique or weather eradicate screwworms (Diptera: Calliphoridae) from Libya?. J. Med. Entomol..

[58] Foil L.D., Hogsette J.A. (1994). Biology and control of tabanids, stable flies and horn flies. Rev. Sci. Tech. OIE.

[59] Ghosh S., Azhahianambi P., Yadav M.P. (2007). Upcoming and future strategies of tick control: A review. J. Vector Borne Dis..

[60] World Organization for Animal Health (OIE) (2011). Terrestrial Animal Health Code—General Provisions.

[61] Knipling E.F. (1938). Internal treatment of animals with phentothiazine to prevent development of horn fly larvae in the manure. J. Econ. Entomol..

[62] Bushland R.C. (1940). The Toxicity of Phenothiazine and Certain Related Compounds to Young Screwworms. J. Econ. Entomol..

[63] Bushland R.C. (1940). The Toxicity of Some Organic Compounds to Young Screwworms. J. Econ. Entomol..

[64] Lindquist A.W., Knipling E.F., Jones H.A., Madden A.H. (1944). Mortality of bedbugs on rabbits given oral dosages of DDT and pyrethrum. J. Econ. Entomol..

[65] Knipling E.F., Bushland R.C., Babers F.H., Culpepper G.H., Raun E.S. (1948). Evaluation of selected insecticides and drugs as chemotherapeutic agents against external bloodsucking parasites. J.Parasitol..

[66] McGregor W.S., Radeleff R.D., Bushland R.C., Claborn H.V. (1955). Dieldrin, aldrin, and lindane as systemic insecticides against livestock pests. Agric. Chem..

[67] Drummond R.O. (1960). Preliminary Evaluation of Animal Systemic Insecticides. J. Econ. Entomol..

[68] McGregor W.S., Bushland R.C. (1956). Research on the Use of Systemic Insecticides for the Control of Livestock Pests. J. Econ. Entomol..

[69] Brundrett H.M., Graham O.H. (1958). Bayer 21/199 as a deterrent to screw-worm attack in sheep. J. Econ. Entomol..

[70] Graham O.H., Moore B., Wrich M.J., Kunz S., Warren J.W., Drummond R.O. (1959). A comparison of ronnel and Co-ral sprays for screwworm control. J. Econ. Entomol..

[71] Wrich M.J., Bushland R.C. (1960). Screwworm control with insecticide sprays. J. Econ. Entomol..

[72] Wrich M.J., Chamberlain W.F., Smith C.L. (1961). Toxicity of General Chemical Compounds 3582, 3583, and 4072 to screw-worms in laboratory and field test. J. Econ. Entomol..

[73] Gladney W.J. (1976). Field Trials of Insecticides in Controlled-release Devices for Control of the Gulf Coast Tick and Prevention of Screwworm in Cattle. J. Econ. Entomol..

[74] World Organization for Animal Health (OIE) (2008). TerrestrialManual—Manual of Diagnostic Tests and Vaccines for Terrestrial Animals.

[75] Wrich M.J. (1961). A Comparison of Co-Ral, Ronnel, and Ruelene Dusts for Screw-worm Control. J. Econ. Entomol..

[76] Drummond R.O., Ernst S.E., Trevino J.L., Graham O.H. (1967). Control of Larvae of the Screwworm in Cattle with Insecticidal Sprays. J. Econ. Entomol..

[77] Bushland R.C., Radeleff R.D., Drummond R.O. (1963). Development of Systemic Insecticides for Pests of Animals in the United States. Annu. Rev. Entomol..

[78] Silva N.M., Azeredo-Espin A.M.L. (2009). Investigation of mutations associated with pyrethroid resistance in populations of the New World Screwworm fly, *Cochliomyia hominivorax* (Diptera: Calliphoridae). Genet. Mol. Res..

[79] Snyder D.E., Lowe L.B., Hacket K.C., Rothwell J.T., Arantes G., Perez-Montie H., Mah C.K. Efficacy of a spinosad aerosol spray formulation against Old and New World screwworm infestations in cattle. Proceedings of the 20th International Conference of the World Association for the Advancement of Veterinary Parasitology.

[80] Ahrens E.H., Gladney W.J., McWhorter G.M., Deer J.A. (1977). Prevention of Screwworm Infection in Cattle by Controlling Gulf Coast Ticks with Slow Release Insecticide Devices. J. Econ. Entomol..

[81] Usher C.B., Cruz J., Carvalho L., Bridi A., Farrington D., Barrick R.A., Eagleson J. (1997). Prophylactic use of ivermectin against cattle myiasis caused by *Cochliomyia hominivorax* (Coquerel, 1858). Vet. Parasitol..

[82] Anziani O.S., Lorefice C. (1993). Prevention of cutaneous myiasis caused by screwworm larvae (*Cochliomyia hominivorax*) using ivermectin. J. Vet. Med..

[83] Anziani O.S., Guglielmone A.A., Aguirre D.H. (1996). Larvicidal activity of abamectin against natural *Cochliomyia hominivorax* larvae infestation. Ann. N Y Acad. Sci..

[84] Anziani O.S., Guglielmone A.A., Aguirre D.H. (1995). Administracion de abamectina para la prevencion de miasis (*Cochliomyia hominivorax*) post castracion en bovinos. Vet. Argent..

[85] Muñiz R.A., Anziani O.S., Ordoñez J., Errecalde J., Moreno J., Rew R.S. (1995). Efficacy of doramectin in the protection of neonatal calves and post-parturient cows against field strikes of *Cochliomyia hominivorax*. Vet. Parasitol..

[86] Muñiz R.A., Coronado A., Anziani O.S., Sanavria A., Moreno J., Errecalde J., Goncalves L.C.B. (1995). Efficacy of injectable doramectin in the protection of castrated cattle against field infestations of *Cochliomyia hominivorax*. Vet. Parasitol..

[87] Anziani O.S., Flores S.G., Moltedo H., Derozier C., Guglielmone A.A., Zimmermann G.A., Wanker O. (2000). Persistent activity of doramectin and ivermectin in the prevention of cutaneous myiasis in cattle experimentally infested with *Cochliomyia hominivorax*. Vet. Parasitol..

[88] Caproni L., Umehara O., Goncalves L.C.B., Moro E. (1998). Persistent efficacy of doramectin and ivermectin in the prevention of natural *Cochliomyia hominivorax* infestations in cattle castrated 10 days after treatment. Rev. Bras. Parasitol. Vet..

[89] Lanusse C., Lifschitz A., Virkel G., Alvarez L., Sánchez S., Sutra J.F., Galtier P., Alvinerie M. (1997). Comparative plasma disposition kinetics of ivermectin, moxidectin and doramectin in cattle. J. Vet. Pharmacol. Ther..

[90] Lima W.S., Malacco M.A.F., Bordin E.L., Oliveira E.L. (2004). Evaluation of the prophylactic effect and curative efficacy of fipronil 1% pour on (Topline ®) on post-castration scrotal myiasis caused by *Cochliomyia hominivorax* in cattle. Vet. Parasitol..

[91] Correia T.R., Scotta F.B., Verocaia G.G., Souza C.P., Fernandesa J.I., Meloa R.M.P.S., Vieiraa V.P.C., Ribeiro F.A. (2010). Larvicidal efficacy of nitenpyram on the treatment of myiasis caused by *Cochliomyia hominivorax* (Diptera: Calliphoridae) in dogs. Vet. Parasitol..

[92] Wright J.E., Smalley H.E., Younger R.L., Crookshank H.R. (1974). Effects of 2 Juvenile Hormone Analogues Against the Screwworm, *Cochliomyia hominivorax* (Coquerel), in Vitro and in Infested Bovine Host. J. Med. Entomol..

[93] Crystal M.M. (1978). Diflubenzuron-induced Decrease of Egg Hatch of Screwworms (Diptera:Calliphoridae). J. Med. Entomol..

[94] Anziani O.S., Guglielmone A.A., Schmid H. (1998). Efficacy of dicyclanil in the prevention of screwworm infestation (*Cochliomyia hominivorax*) in cattle castration wounds. Vet. Parasitol..

[95] Knipling E.F. (1942). Acquired Resistance to Phenothiazine by Larvae of the Primary Screwworm. J. Econ. Entomol..

[96] Rawlins S.C., Whitten C.J., McInnis D.O. (1983). Survey of Resistance to Insecticides among Screwworm (Diptera: Calliphoridae) Populations from Various Geographical Regions. J. Econ. Entomol..

[97] Carvalho R.A., Azeredo-Espin A.M.L., Torres T.T. (2010). Deep sequencing of New World screwworm transcripts to discover genes involved in insecticide resistance. BMC Genomics.

[98] Tannahill F.H. (1978). Development of an Adult Suppression System for the Screwworm Fly, *Cochliomyia hominivorax* (Coquerel). M.S. thesis.

[99] Peterson II R.D., Gagne R.J., Snow J.W., Spencer J.P. (1981). Attraction of non-target organisms to SWASS. Environ. Entomol..

[100] Coppedge J. R., Goodenough J.L., Broce A.B., Tannahill F.H., Snow J.W., Crystal M.M., Petersen H.D. (1978). Evaluation of the Screwworm Adult Suppression System (SWASS) on the Island of Curacao. J. Econ. Entomol..

[101] Goodenough J.L., Coppedge J.R., Broce A.B., Petersen H.D., Higgins A. (1979). A System for Baiting and Distributing Screwworm Adult Suppression System Units. Transactions of the A.S.A.E.

[102] Coppedge J.R., Brown H.E., Goodenough J.L., Tannahill F.H., Snow J.W., Petersen H.D., Hofmann H.C. (1980). Field Performance of a New Formulation of the Screwworm Adult Suppression System. J. Econ. Entomol..

[103] Tannahill F.H., Coppedge J.R., Wendel L.E., McInnis D.O., Petersen H.D. (1982). Evaluation of the Screwworm Adult Suppression System (SWASS) on the Pacific Coast of Mexico. Southwest. Entomol..

[104] Mackley J.W., Brown H.E. (1987). Efficacy of Different-sized Pellets of the Screwworm (Diptera: Calliphoridae) Adult Suppression System (SWASS) During the Wet and Dry Seasons in Mexico. J. Econ. Entomol..

[105] Peterson II R.D., Coppedge J.R., Brown H.E., Del Var Petersen H. (1983). Evaluation of a New Insecticide for Use in Screwworm Adult Suppression System Pellets, *Cochliomyia hominivorax*. J. Econ. Entomol..

[106] Coppedge J.R., Brown H.E., Snow J.W., Tannahill F.H. (1981). Bait Stations for the Suppression of Screwworm Populations. J. Econ. Entomol..

[107] FAO (2005). Glossary of Phytosanitary Terms. Provisional Additions.

[108] Melvin R., Bushland R.C. (1936). A method of rearing Cochliomyia americana C. and P. on artificial media. Technical report for Bureau of Entomology and Plant Quarantine.

[109] Brown H.E., Snow J.W. (1979). Screwworms (Diptera: Calliphoridae): A new liquid media for rearing screwworm larvae. J. Med. Entomol..

[110] Harris R.L., Gersabeck E.F., Corso C., Graham O.H. (1985). Screwworm larval production on gelled media. Southwest. Entomol..

[111] Taylor D.B., Mangan R.L. (1987). Comparison of gelled and meat diets for rearing screwworm, *Cochliomyia hominivorax* (Diptera: Calliphoridae) larvae. J. Econ. Entomol..

[112] Taylor D.B., Bruce J.C., Garcia R. (1991). Gelled diet for screwworm (Diptera: Calliphoridae) mass production. J. Econ. Entomol..

[113] Chaudhury M.F., Skoda S.R. (2007). A cellulose fiber-based diet for screwworm (Diptera: Calliphoridae) larvae. J. Econ. Entomol..

[114] Brewer F.D. (1993). Meridic Diets for Adult Screwworm (Diptera: Calliphoridae). J. Entomol. Sci..

[115] Hammack L. (1999). Stimulation of oogenesis by proteinaceous adult diets for screwworm, *Cochliomyia hominivorax* (Diptera: Calliphoridae). Bull. Entomol. Res..

[116] Chaudhury M.F., Alvarez L.A., Velazquez L.L. (2000). A meatless diet for adult screwworm (Diptera: Calliphoridae). J. Econ. Entomol..

[117] Smith C.L. (1960). Mass Production of Screw-worms *Callitroga hominivorax* for the Eradication Program in the Southeastern States. J. Econ. Entomol..

[118] Crystal M.M. (1965). First Efficient Chemosterilants Against Screw-worm Flies (Diptera:Calliphoridae). J. Med. Entomol..

[119] Crystal M.M. (1971). Sexual Sterilization of Screwworm Flies: Further Studies of Reliability of the Chemosterilant Technique. J. Med. Entomol..

[120] Bakri A., Mehta K., Lance D.R., Dyck V.A., Hendrichs J., Robinson A.S. (2005). 2005. Sterilizing insects with ionizing radiation. Sterile Insect Technique: Principles and Practice in Area-wide Integrated Pest Management.

[121] Muller H.J. (1927). Artificial transmutation of the gene. Science.

[122] Bushland R.C., Hopkins D.E. (1951). Experience with screwworm flies sterilized by X-rays. J. Econ. Entomol..

[123] Lindquist A.W. (1955). The Use of Gamma Radiation for Control or Eradication of the Screw-worm. J. Econ. Entomol..

[124] Bushland R.C., Hopkins D.E. (1953). Sterilization of screwworm flies with X-rays and gamma rays. J. Econ. Entomol..

[125] Baumhover A.H. (1963). Influence of aeration during gamma irradiation of screwworm pupae. J. Econ. Entomol..

[126] Crystal M.M. (1979). Sterilization of screwworm flies (Diptera: Calliphoridae) with gamma rays: restudy after two decades. J. Med. Entomol..

[127] Mastrangelo T., Parker A.G, Jessup A., Pereira R., Orozco-Dávilla D., Islam A., Dammalage T., Walder J.M. (2010). A new generation of X ray irradiators for insect sterilization. J. Econ. Entomol..

[128] Baumhover A.H. (1966). Eradication of the Screwworm Fly, an Agent of Myiasis. J. Am. Med. Assoc..

[129] Wyss J.H., Tan K.H. (2000). Screw-worm eradication in the Americas – overview. Area-wide control of Fruit Flies and Other Insect Pests.

[130] Garcia R., Mendez L., Serrano E., Morales T.G., Vreysen M.J.B., Vreysen M.J.B., Robinson A.S., Hendrichs J. (2007). Insecticidal wound treatment of livestock on Isla de la Juventud, Cuba: an efficient suppression method of new world screwworm *Cochliomyia hominivorax* prior to the release of sterile insects. Area-wide Control of Insect Pests: From Research to Field Implementation.

[131] Hendrichs J., Vreysen M.J.B., Enkerlin W.R., Cayol J.P., Dyck V.A., Hendrichs J., Robinson A.S. (2005). Strategic options in using sterile insects for Area-Wide Integrated Pest Management. Sterile Insect Technique: Principles and Practice in Area-wide Integrated Pest Management.

[132] Vreysen M.J.B., Gerardo-Abaya J., Cayol J.P., Vreysen M.J.B., Robinson A.S., Hendrichs J. (2007). Lessons from area-wide integrated pest management (AW-IPM) programmes with an SIT component: An FAO/IAEA perspective. Area-wide Control of Insect Pests: From Research to Field Implementation.

[133] Mastrangelo T., Chaudhury M.F., Skoda S.R., Welch J.B., Sagel A., Walder J.M.M.  (2012). Feasibility of Using a Caribbean Screwworm for SIT Campaigns in Brazil. J. Med. Entomol..

